# MAP7 promotes migration and invasion and progression of human cervical cancer through modulating the autophagy

**DOI:** 10.1186/s12935-020-1095-4

**Published:** 2020-01-13

**Authors:** Li Zhang, Xudong Liu, Lina Song, Hui Zhai, Chaohua Chang

**Affiliations:** 1 Department of Gynaecology and Obstetrics, Jinan Women and Children Health Hospital, No. 2 Jianguo Xiaojing 3rd Road Center Area, Jinan, Shandong 250001 China; 2 Department of Pain, Qilu Hospital of Shandong University, Jinan, China; 3 Department of Anethesia, Jinan Women and Children Health Hospital, No. 2 Jianguo Xiaojing 3rd Road Center Area, Jinan, Shandong 250001 China

**Keywords:** Cervical cancer, MAP7, Migration and invasion, Epithelial-mesenchymal transition

## Abstract

**Background:**

Microtubule-associated proteins 7(MAP7) was reported to be engaged into the function of neuronal function. The function of MAP7 in human cervical cancer (CC) was unknown. We aimed to uncover the function and mechanism of MAP7 on CC.

**Methods:**

We applied qRT-PCR, western blot and immunochemistry to detect the expression difference between normal tissue and CC. In vitro, we establish MAP7 stable knocking down and overexpression cell lines and investigated the function and underlying mechanism of MAP7 in CC.

**Results:**

Both mRNA and protein of MAP7 were upregulated in CC compared with the normal tissue. MAP7 was correlated with the clinical stage and tumor size and lymph node metastasis. MAP7 promotes the invasion and migration of CC cell lines. We next detected EMT pathway and autophagy associated pathway. MAP7 promotes the EMT through modulating the autophagy.

**Conclusion:**

Taken above, our results showed that MAP7 promotes the migration and invasion and EMT through modulating the autophagy.

## Background

Cervical cancer (CC) is the second most common gynecological carcinoma worldwide [1]. Through a lot of advancements in radiotherapy and immunotherapy was applied. Many patients with late stage of CC still suffers from recurrence and metastasis [2, 3]. However, more biomarkers predicting the prognosis and therapy targets were still needed.

Microtubule was responsible for vital cellular processes such as cell division and differentiation [4, 5]. Microtubules are continue remodeled through alternating growth and shrinkage of their extremities, known as dynamic instability. Microtubule associated protein 7 (MAP7) was crucial in the regulating of microtubule bundling and dynamics. MAP7 was reported to be engaged into the function of neuron cell [6]. MAP7 was reported to be upregulated and correlated with the prognosis of cancers [7, 8]. The function and mechanism of MAP7 in CC was still unknow.

We applied qRT-PCR, Western blot and immunochemistry to detect the expression difference between normal tissue and CC after examining the function of MAP7 on CC cell lines. We found that MAP7 promotes the EMT of CC cell line through modulating the autophagy.

## Materials and methods

### Tissue samples

All human tissues were obtained from the Department of Gynaecology and Obstetrics, Qilu Hospital of Shandong University and Department of Gynaecology and Obstetrics, Jinan Women and Children Health Hospital after confirmation by a pathologist. Tissues were obtained with the patients’ written and informed consent approved by the institution’s Institutional Review Board of Qilu Hospital of Shandong University and Department of Gynaecology and Obstetrics.

### Immunohistochemistry

Samples were fixed in 10% neutral buffered formalin for 7 days, embedded in paraffin and sectioned. Slides were baked, deparaffinization, blocked and incubated with anti-MAP7 (1:100 dilution, CST) overnight at 4 °C. After washing, the samples were treated with corresponding secondary antibody, Colour was developed using a DAB (3,3′-diaminobenzidine) Horseradish Peroxidase Color Development Kit (Beytime).

### Cell culture

The normal cervical epithelial ECT cell line and the human CC cell lines were obtained from American Type Culture Collection (Manassas, VA, USA). SiHa HeLa229 and MS751 cells were cultured in DMEM (Sigma-Aldrich, St. Louis, MO, USA) with 10% foetal bovine serum (FBS; Invitrogen, Carlsbad, CA, USA) and penicillin–streptomycin, whereas other cell lines were cultured in DMEM F-12 medium containing 10% FBS with 1% penicillin–streptomycin.

### Real-time (RT) qPCR

Total RNA from cells or tissues was extracted using TRIzol reagent (Thermo Fisher Scientific, Waltham, MA, USA) according to the manufacturer’s protocol. Quantitative RT (qRT)-PCR was conducted using SYBR Green PCR Master Mix (Takara Bio) according to the protocol. The quantification was applied on a CFX96 Real-Time PCR Detection System (Bio-Rad, Hercules, CA, USA). Relative expression was normalized to Actin.

### Western blot assay

Equal amounts of protein extracts were run on 12% SDS-PAGE and after trans-membrane and blocking, it was incubated with a primary antibody overnight at 4 °C. Finally, the membrane was incubated with its respective horseradish peroxidase-conjugated secondary antibodies for 1 h at room temperature. The protein bands of interest were visualized using enhanced chemiluminescence reagents (Millipore, Burlington, MA, USA).

### Trans-well assay

Trans-well assay was applied to measure the migration and invasion ability. Certain number of 7 × 10^4^ cells/well were resuspended in 250 μL of plain medium without any FBS in the upper chamber (8-μm pore size, Costar, Corning, NY, USA) and the lower chamber was filled with 0.75 mL of medium supplemented with 10% FBS. After incubation for 24 h at 37 °C, the invasive and migrated cells were fixed with 100% methanol and stained with 0.5% crystal violet for 20 min before counting under an inverted microscope.

### Wound healing assay

We performed a wound healing assay to detect the migration ability of cells. We seeded 1 × 105 cells per well into a 6-well plate. Cells were incubated with complete medium (10% FBS and 1% PS with corresponding medium mentioned above). After the cells reached 100% confluency, equal wounds were made with the 1 ml-pipette. After incubating for 24 h. The relative width was measured by the ration of area and length. The area was calculated through ImageJ following the protocol automatically.

### Lentiviral production and stable cell line construction

Lentiviral vectors expressing shRNA and MAP7 were obtained from Bio-Chemistry China. CC cells were then transduced by the above lentiviruses with polybrene (8 mg/ml, Sigma). After incubating for 24 h, cells were selected with 2 mg/ml puromycin for 3 days. Stable cell lines were harvested.

### Statistical analysis

Statistical analyses were performed using SPSS version 18.0 (SPSS Inc., Chicago, IL, USA). Data are presented as the mean ± SD. The Chi squared test was used to analyse clinicopathological characteristics. Comparisons between two groups were evaluated using Student’s *t* test. Differences among the groups were tested using a one-way ANOVA followed by Turkey post test. All experiments were repeated at least three times. A p value < 0.05 was considered statistically significant.

## Results

### MAP7 was upregulated in cervical cancer

**Fig. 1:**
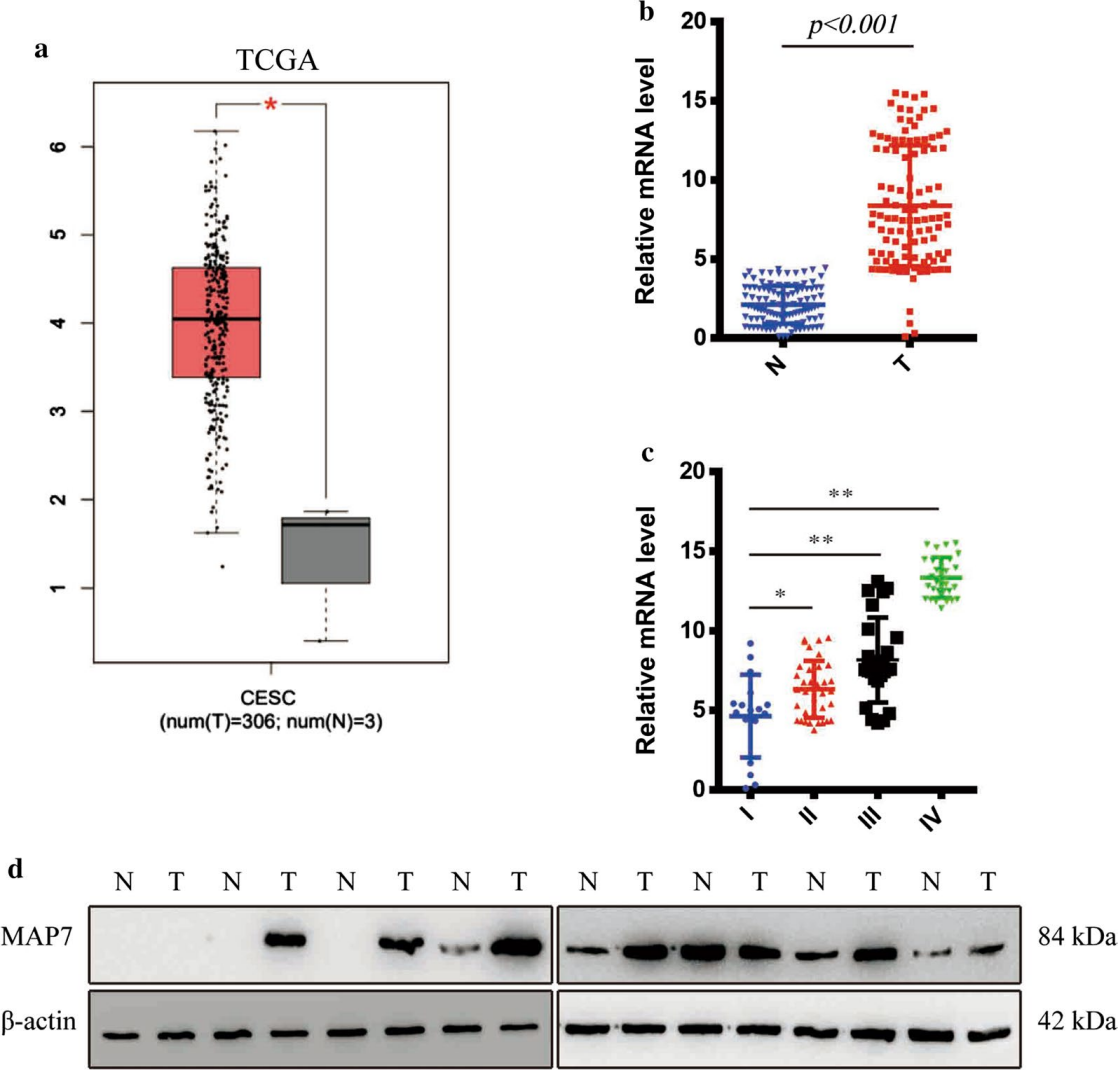
MAP7 was upregulated in CC tissues. **a** The relative level of MAP7 of CC and paired normal tissue in TCGA database. **b** The relative level of MAP7 of CC in our own database. **c** The relative level of MAP7 in different stages of CC. **d** Western blot of MAP7 in 8 paired CC and normal tissue

We scanned the most authoritative database in cancer-TCGA database and identified MAP7 was upregulated in 306 Cervical cancer compared with normal tissue (Fig. 1a). The number of normal tissues in TCGA database was small so we detected the mRNA level of MAP7 in 105 CC tissues and paired normal tissues mRNA level of MAP7 was upregulated in CC tissue (p < 0.001) (Fig. 1b). The clinical features of the 105 patients was shown in Table 1. We next analysed the correlation of MAP7 and the clinical stage of Cervical cancer, we next divided the whole cohort into 4 groups according to the clinical stage. the level of MAP7 was correlated with the clinical stage. The mRNA level of MAP7 was highest in stage IV while the mRNA level of MAP7 in stage I was the lowest (Fig. 1c). We next detected the protein level of MAP7 in 8 patients. The protein level of MAP7 increased in 6 out of 8 patients (Fig. 1d).

**Table 1:** The clinical characteristic of patients in our own database

Characteristics	Patients (n = 105)	MAP7 lower (n = 31)	MAP7 higher (n = 74)	p value
Age (years)				
< 55	51	38	13	> 0.05
≥ 55	54	36	18	
Stage				
I + II	42	24	18	< 0.01
III + IV	63	7	56	
Size (cm)				
< 4	43	16	27	< 0.01
≥ 4	62	15	47	
Lymph node				
Negative	54	20	34	< 0.01
Positive	51	11	40	

### MAP7 was correlate with the prognosis

**Fig. 2:**
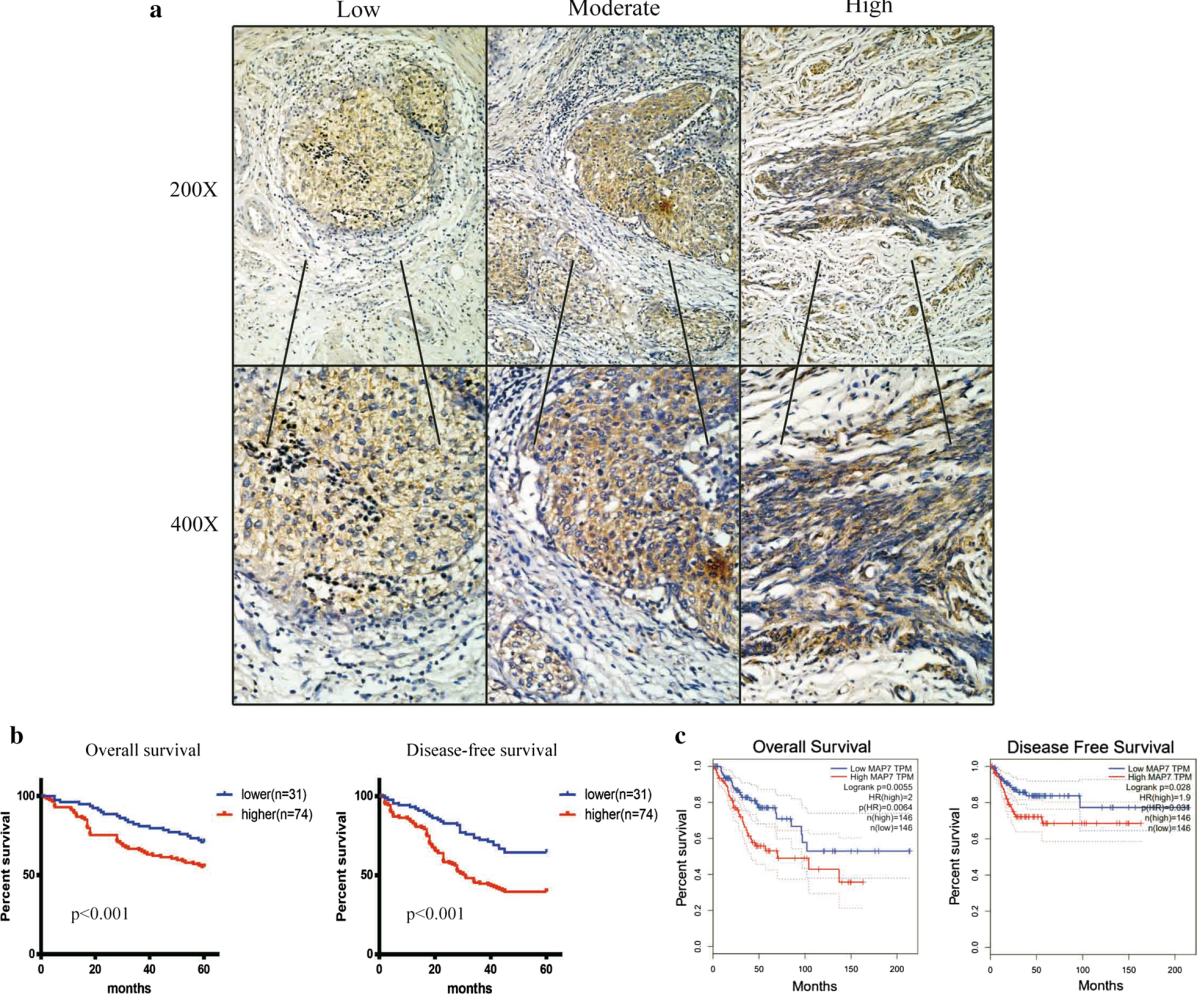
MAP7 was correlated with the prognosis. **a** The representative image of IHC of CC tissue. **b** Left: the overall survival analysis of patients of our own database right: the disease free survival analysis of patients of our own database. **c** Left: the overall survival analysis of TCGA database right: the disease free survival analysis of TCGA database

We applied IHC and performed the survival analysis according to the expression of MAP7(Fig. 2a). MAP7 was corelated with the overall survival time (OS) and disease-free survival time (DFS). Patients with higher level of MAP7 has shorter OS and DFS in our own database (Fig. 2b). We next scanned the TCGA database and found that MAP7 was upregulated in tumour and negative correlated with OS and DFS (Fig. 2c).

### Stable cell line establishment

**Fig. 3:**
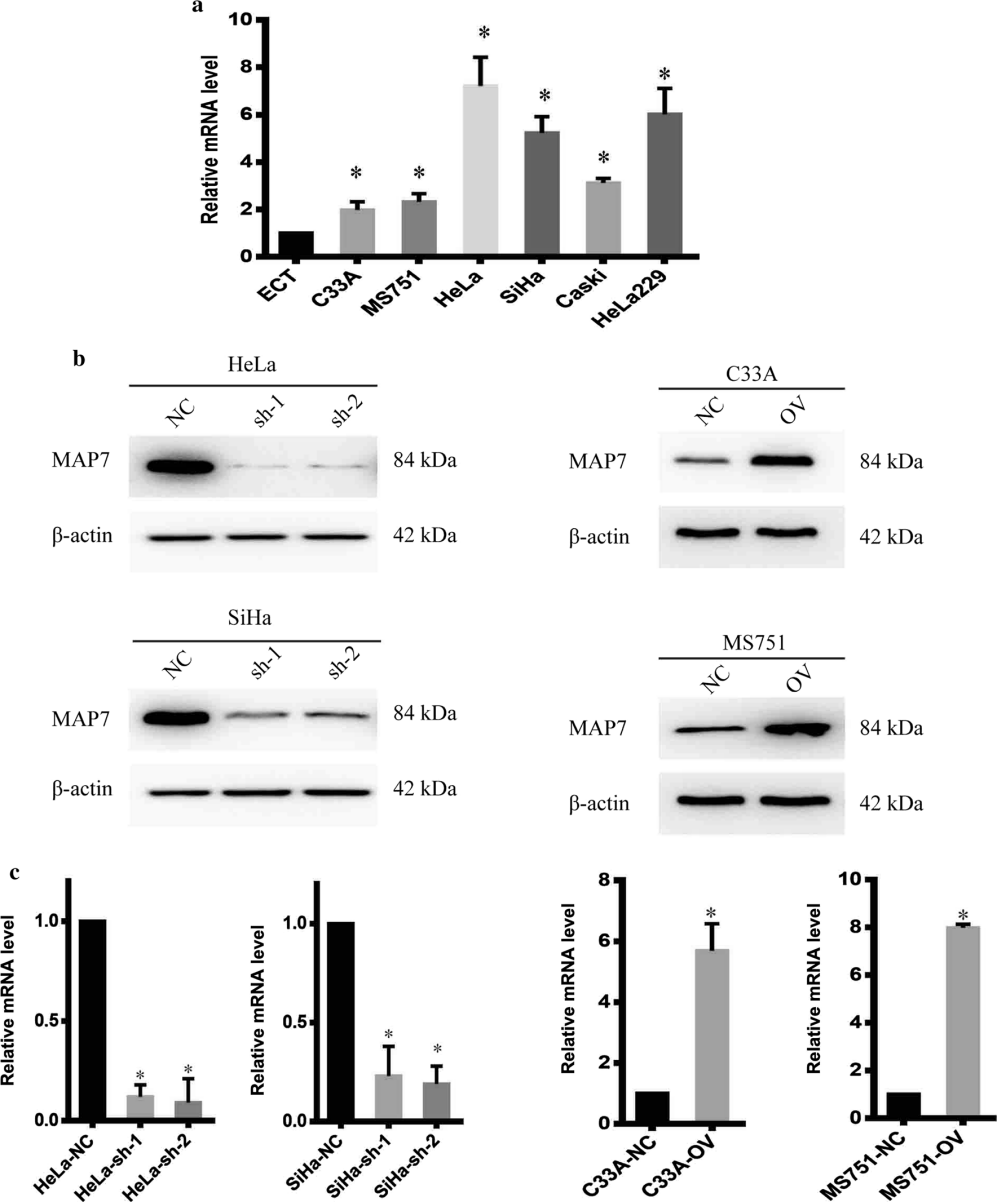
Stable cell line was established. **a** The relative mRNA level of MAP7 in different cell lines. **b** Western blot of MAP7 of different cell lines. **c** The relative mRNA level of MAP7 of different cell lines

To uncover the biological function of MAP7, we detect the mRNA level of MAP7 in cervical cancer cell lines. MAP7 was upregulated in CC cell line. HeLa and SiHa possessed the higher level of MAP7 while C33A and MS751 possessed the lowest level of MAP7 (Fig. 3a). We next establish stable cell line with lentivirus. The mRNA and protein level of MAP7 was shown in Fig. 3b, c.

### MAP7 promotes the migration and invasion of CC cell

**Fig. 4:**
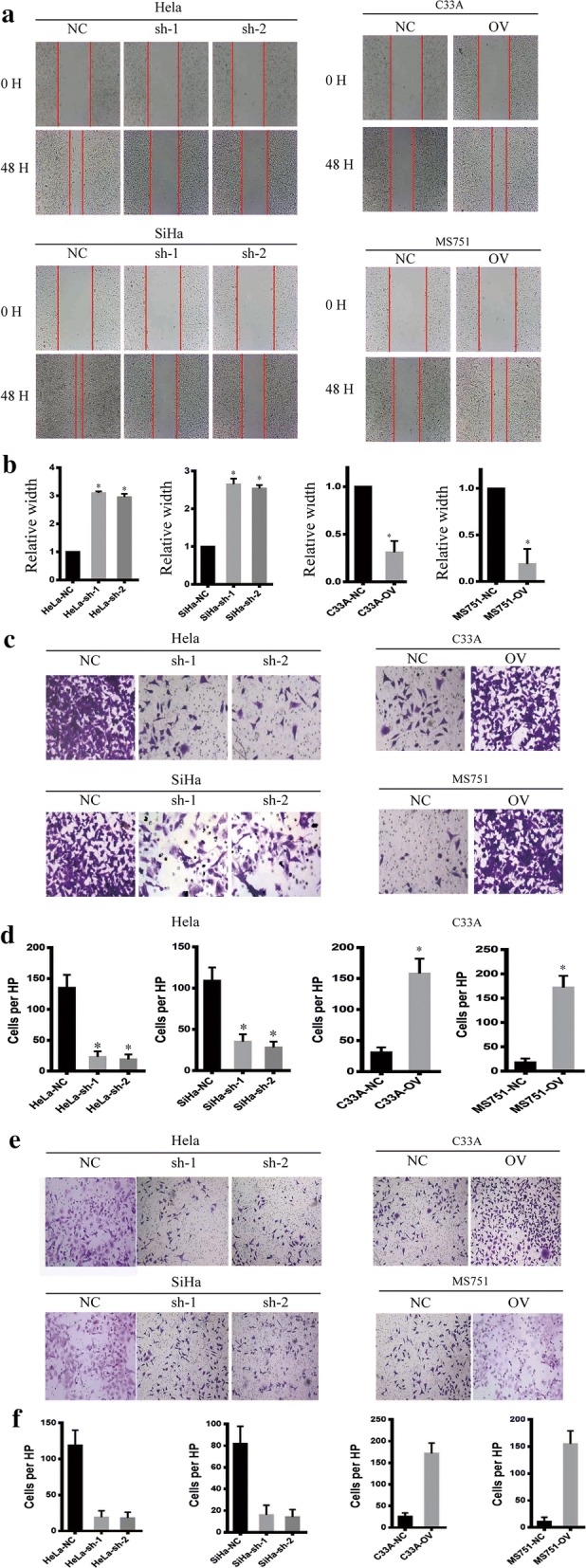
Map7 promotes the migration and invasion of CC. **a** The representative image of wounding healing. **b** The statistical analysis of wound healing. **c** The representative image of trans-well. **d** The statistical analysis of trans-well. **e** The representative image of invasion chamber. **f** The statistical analysis of invasion chamber

MAP7 was shown to be negatively correlated with prognosis as previously described. However, the potential function was still unknown, to uncover the biological function of MAP7 on CC cells, we next examined the function of MAP7 on migration and invasion as recurrence and metastasis were the most reasons for patient-death. We applied the wound healing assay, the Trans-well assay and the invasion chamber assay to measure the migrate ability. The wound healed more rapidly in the MAP7-overexpressing cell line than in the MAP7 knockdown cell line (Fig. 4a, b), the MAP7-overexpressing cells harbour improved migrate ability than the MAP7 knockdown cells in the Trans-well assay (Fig. 4c, d) as well as the invasion chamber assay (Fig. 4e, f). Taken above data, MAP7 promotes the migration and invasion of CC.

### MAP7 promotes the EMT of CC

**Fig. 5:**
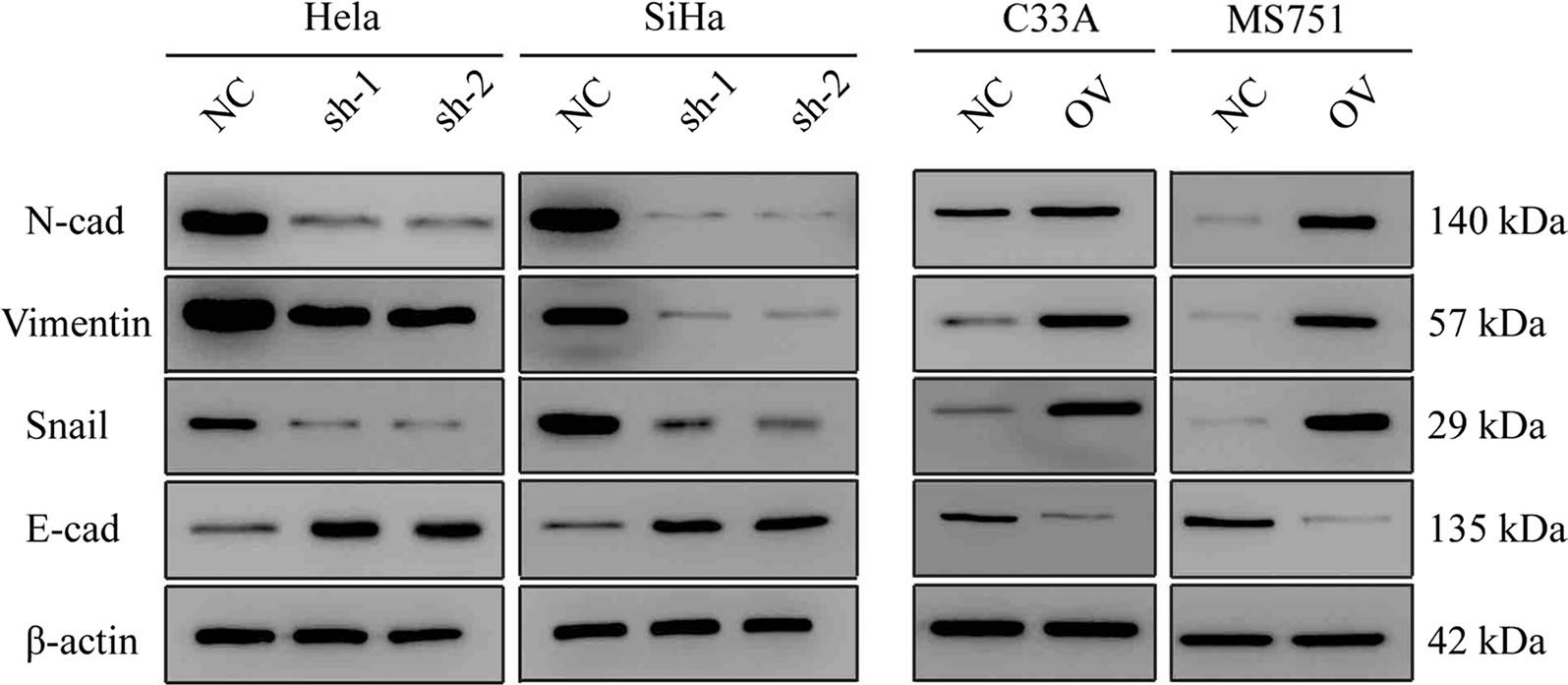
Map7 promotes the EMT of CC western blot of EMT markers of CC cell line

We have proved that MAP7 promotes migration and invasion in CC. EMT markers are the most common markers reflecting cell mesenchymal and epithelial status. We detected the expression pattern of classic EMT markers. Mesenchymal markers such as N-cadherin, Snail and Vimentin decreased in MAP7 knocking down cells, but epithelial markers such as E-cadherin increased in the shMAP7 cell line; however, mesenchymal markers increased but epithelial markers decreased at both the RNA and protein level in the MAP7-overexpressing cell line (Fig. 5), which coordinates with the former results.

### MAP7 promotes the cell cycle through modulating the autophagy

**Fig. 6:**
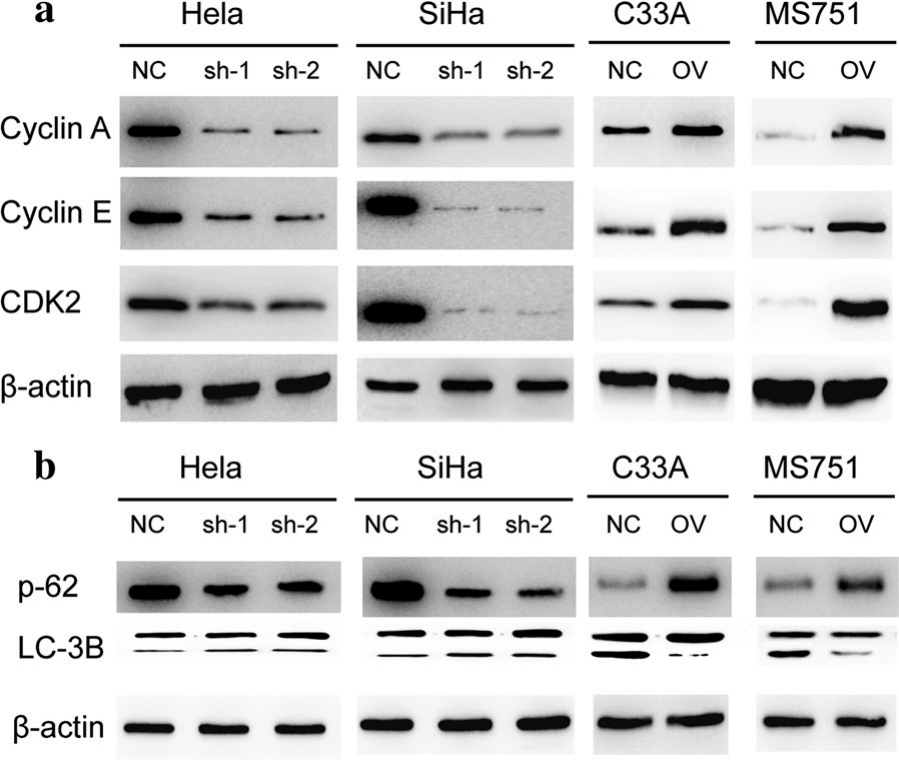
Map7 modulates the cell cycle and the autophagy pathway. **a** Western blot of cell cycle check point of CC cell line. **b** Western blot of autophagy related pathway of CC cell line

We have proved that MAPT promotes the migration and invasion in CC through EMT pathway. MAP7 was reported to involve in the autophagy pathway. We next detect the cell cycle check point and the autophagy pathway. The results showed that the MAP7 knocking down resulted in the disturbed cell cycle. The expression level of cell cycle check point decreased in cells with lower level of MAP7 (Fig. 6a). As presented in Fig. 6b, the ratio of LC3B-II/LC-3BI increased with the knocking down of MAP7 and p62 showed the opposite change which indicates that MAP7 modulates the autophagy pathway.

## Discussion

Human cervical cancer was one of the most common malignancy worldwide. Though advancement in radio therapy and immunotherapy, patients with late stage of cervical cancer still suffers from recurrence and metastasis. Therefore, more biomarkers and therapeutic targets were needed.

Microtubule formation was crucial for the mitosis, Microtubules are continually remodeled through alternating growth and shrinkage of their extremities, known as dynamic instability [5, 9]. Microtubule associated protein 7 (MAP7) was crucial in the regulating of microtubule bundling and dynamics. Researches has shown that MAP7 plays a novel role in the formation of microtubule in neuron through directly binding to the microtubule and promotes its’ stabilization [10]. Further domain analysis of MAP7 shows that MAP7 enhances kinesin-1 recruitment to microtubules, both N and P domains are responsible for branch formation, whereas the C-terminal kinesin-interacting domain is important for promoting axon and branch growth and branch growth mediated by the C domain can compete for main axon growth [6]. As the domain of MAP7 exert its’ function independence of full length of MAP7, proteins with the same domain of MAP7 was reported to involve into the formation of microtubule [11]. MAP7 was reported to contribute to the self-renewal of stem cells [12]. However, the role of MAP7 in cancers was rarely studied. Studies showed that MAP7 was upregulated in young patients with cytogenetically normal acute myeloid leukemia and predicts poor outcome [13]. The potential function and mechanism of MAP7 in cancer was still unknown. Hence, we examine the level of MAP7 in cervical cancer and its’ correlation with prognosis. Patients with higher level of MAP7 harbor shorter overall survival time and disease free time. To uncover the biological function of MAP7, we detect the mRNA and protein level of MAP7 in CC cell line and establish knockdown and overexpress cell lines. Cells with higher level of MAP7 migrates and invades more easily than cells with lower level of MAP7. We next detect the mesenchymal markers and epithelial markers in the cells above. MAP7 promotes the transition from epithelial status to mesenchymal status. We next detect the autophagy associated pathway. The results showed that the ratio of LC3B-II/LC-3BI increased with the knocking down of MAP7 and p62 showed the opposite change which indicates that MAP7 modulates the autophagy pathway.

Taken together, our research shows that MAP7 promotes migration and invasion and progression of human cervical cancer through modulating the autophagy pathway. Further specific mechanism MAP7 on cancer cell was still needed to make it an ideal therapeutic target.

## Conclusion

The findings of this study demonstrate that MAP7 is overexpressed in CC tissues is strongly associated with a poor prognosis. MAP7 promotes migration and invasion and progression of human cervical cancer through modulating the autophagy pathway. Therefore, MAP7 can serve as the potential therapeutic target.

## Data Availability

All the datasets supporting the conclusions of this article are included within the article.

## References

[1] Shaitelman SF, Cromwell KD, Rasmussen JC, Stout NL, Armer JM, Lasinski BB, Cormier JN (2015). Recent progress in the treatment and prevention of cancer-related lymphedema. CA Cancer J Clin.

[2] Maresso KC, Tsai KY, Brown PH, Szabo E, Lippman S, Hawk ET (2015). Molecular cancer prevention: current status and future directions. CA Cancer J Clin.

[3] Porcza LM, Simms C, Chopra M (2016). Honey and cancer: current status and future directions. Diseases.

[4] Du L, Risinger AL, Yee SS, Ola AR, Zammiello CL, Cichewicz RH, Mooberry SL (2019). Identification of C-6 as a new site for linker conjugation to the taccalonolide microtubule stabilizers. J Nat Prod.

[5] Camargo OG, Falk S, Johansson PA, Peyre E, Broix L, Sahu SK, Hirst W, Schlichthaerle T, De Juan RC, Draganova K, Vinopal S, Chinnappa K, Gavranovic A, Karakaya T, Steininger T, Merl-Pham J (2019). The centrosome protein AKNA regulates neurogenesis via microtubule organization. Nature.

[6] Tymanskyj SR, Yang BH, Verhey KJ, Ma L (2018). MAP7 regulates axon morphogenesis by recruiting kinesin-1 to microtubules and modulating organelle transport. ELIFE.

[7] Blum C, Graham A, Yousefzadeh M, Shrout J, Benjamin K, Krishna M, Hoda R, Hoda R, Cole DJ, Garrett-Mayer E, Reed C, Wallace M, Mitas M (2008). The expression ratio of Map7/B2M is prognostic for survival in patients with stage II colon cancer. Int J Oncol.

[8] Yan X, Liang H, Deng T, Zhu K, Zhang S, Wang N, Jiang X, Wang X, Liu R, Zen K, Zhang CY, Ba Y, Chen X (2013). The identification of novel targets of miR-16 and characterization of their biological functions in cancer cells. Mol Cancer.

[9] Yadav SK, Stojkov D, Feigelson SW, Roncato F, Simon HU, Yousefi S, Alon R (2019). Chemokine-triggered microtubule polymerization promotes neutrophil chemotaxis and invasion but not transendothelial migration. J Leukoc Biol.

[10] Tymanskyj SR, Yang B, Falnikar A, Lepore AC, Ma L (2017). MAP7 regulates axon collateral branch development in dorsal root ganglion neurons. J Neurosci.

[11] Yadav S, Verma PJ, Panda D (2014). C-terminal region of MAP7 domain containing protein 3 (MAP7D3) promotes microtubule polymerization by binding at the C-terminal tail of tubulin. PLoS ONE.

[12] Gallaud E, Caous R, Pascal A, Bazile F, Gagne JP, Huet S, Poirier GG, Chretien D, Richard-Parpaillon L, Giet R (2014). Ensconsin/Map7 promotes microtubule growth and centrosome separation in Drosophila neural stem cells. J Cell Biol.

[13] Fu L, Fu H, Zhou L, Xu K, Pang Y, Hu K, Wang J, Tian L, Liu Y, Wang J, Jing H, Huang W, Ke X, Shi J (2016). High expression of MAP7 predicts adverse prognosis in young patients with cytogenetically normal acute myeloid leukemia. Sci Rep.

